# Associations between attenuated auditory p300 event‐related potential and cognitive basic symptoms in young people at clinical high risk for psychosis

**DOI:** 10.1111/pcn.13886

**Published:** 2025-08-21

**Authors:** James C. Martin, K. Oliver Schubert, Daniel H. Mathalon, Simon Hartmann, Scott R. Clark

**Affiliations:** ^1^ Discipline of Psychiatry, Adelaide Medical School The University of Adelaide Adelaide South Australia Australia; ^2^ Division of Mental Health, Northern Adelaide Local Health Network SA Health Adelaide South Australia Australia; ^3^ Headspace Early Psychosis Sonder Adelaide South Australia Australia; ^4^ Department of Psychiatry and Behavioral Sciences University of California San Francisco California USA; ^5^ San Francisco Veterans Affairs Medical Center San Francisco California USA; ^6^ UCSF Weill Institute for Neurosciences San Francisco California USA; ^7^ Centre for Youth Mental Health The University of Melbourne Melbourne Victoria Australia; ^8^ Orygen Melbourne Victoria Australia; ^9^ Basil Hetzel Institute Woodville South Australia Australia

**Keywords:** basic symptoms, clinical‐high‐risk, COGDIS, electroencephalography, P300

## Abstract

**Aims:**

The value of assessing basic symptoms in clinical‐high‐risk for psychosis (CHR) is becoming increasingly apparent. Greater recognition of subjective experience in neuroscience and psychiatry has renewed research interest in electrophysiological biomarkers of basic symptoms. This study aims to investigate whether cognitive basic symptoms (COGDIS), which capture a subset of basic symptoms, are associated with P3b attenuation and the modulation of brain connectivity in a large sample of CHR.

**Methods:**

Data from the North American Prodromal Longitudinal Study‐ 3 (NAPLS‐3) comprised 440 male and female CHR individuals who completed both the COGDIS items of the schizophrenia proneness instrument as well as a two‐tone auditory oddball task. P3b amplitude was measured at the central (Cz) as well as left (P3) and right (P4) parietal electrodes. Brain connectivity was calculated across 300 ms windows before (−300 ms to 0 ms) and after (100 ms to 400 ms) onset of target stimuli. Brain connectivity modulation was calculated as the difference between pre‐stimulus and post‐stimulus windows.

**Results:**

Multiple linear regression analysis indicated that COGDIS was associated with reduced P3b amplitude at the P4 electrode. This effect was not associated with the severity of positive or negative symptoms. No differences in connectivity strength or modulation were found between the groups.

**Conclusions:**

In a large sample of CHR/UHR individuals, cognitive basic symptoms criteria was associated with reduced P3b amplitude at the P4 electrode, approximating the right temporo‐parietal area. Parietal P3b attenuation may reflect greater preoccupation towards sensory data, which could play a role in cognitive basic symptom pathogenesis.

Within modern neuroscience and psychiatry, the value of subjective experiences as expressions of specific neurophysiological processes is becoming increasingly recognized.^1^ One example, Huber's basic symptoms concept, refers to subtle subjective alterations of self‐experience, such as volition and thought, which indicate vulnerability for future psychosis. Often detectable prior to recognizable functional decline, the use of basic symptoms as a screening tool for early psychosis originates from the Cologne Early Recognition Study,^2^ where a subset of basic symptoms predicted psychosis‐risk, later operationalized within the cognitive disturbances (COGDIS) criteria. The Clinical‐High Risk (CHR) / Ultra‐High Risk (UHR) approaches represent a separate method of screening for psychosis‐risk. Adopted internationally,^3^ the scales capture those exhibiting recent decline in function, then stratify subjects into at‐risk groups according to family history and severity of positive, negative, and disorganized symptoms.^4^ A substantial proportion of individuals who transition to psychosis screen negative for CHR/UHR criteria at cross‐sectional assessment,^5^ whilst for those who screen positive, more than three quarters report a pre‐existing comorbid diagnosis, with both personality and neurodevelopmental disorders commonly co‐occurring.^6^

Two recent large‐sample international studies have demonstrated the value of assessing basic symptoms in individuals with CHR. Firstly, in the third phase of the North American Prodrome Longitudinal Study (NAPLS‐3), COGDIS was prevalent across common comorbidities in CHR, but almost twice as prevalent in schizotypal personality disorder, as opposed to autism spectrum disorder or borderline personality disorder.^7^ Moreover, the rate of transition was twice as high in the COGDIS positive group. A separate latent class analysis of a broad range of clinical features in a sample of over 800 CHR participants from specialized early intervention centers for psychosis in Germany and Switzerland found that basic symptoms best differentiated between the three identified clinical classes, with a transition rate of 55% in the ‘high‐symptom’ group.^8^

A greater appreciation of phenomenology in emerging psychosis has renewed research interest in the neurophysiology underlying basic symptoms. Current biomarker studies primarily originate from neuroimaging and neurophysiology methods, including magnetic resonance imaging and electroencephalography (EEG).^9^, ^10^ Etkin and Mathalon^11^ advocate for the use of EEG over structural or functional magnetic resonance imaging, claiming that scalability, cost‐effectiveness, and reliability establishes EEG as most likely to have clinical utility in the near future. Simultaneously, they acknowledged the need for larger samples to improve the translation into clinical practice as small sample sizes are a common limitation of research on biomarkers for basic symptoms.^11^

Two commonly referenced electrophysiological candidates for dysfunction in CHR/UHR are the mismatch negativity and P300 event related potentials (ERP). Mismatch negativity describes a negative signal deflection around 100–250 ms after a deviant stimulus, whereas the P300 ERP component is a positive signal deflection about 300ms after stimulus onset. In schizophrenia and CHR populations, lower mismatch negativity and P300 amplitude is often reported relative to controls and these findings have been replicated many times.^12^, ^13^ According to a review of basic symptoms and electrophysiology, no relationship exists between mismatch negativity and basic symptoms or COGDIS in CHR.^9^ Similarly, Nelson, Lavoie^14^ found no association between mismatch negativity and the related concept of Self‐Disorder,^15^ which was developed from the basic symptoms concept and later operationalized in the 57‐item examination of anomalous self‐experience.^16^ In contrast, CHR participants who endorsed basic symptoms items, recorded greater P300 attenuation at temporo‐parietal electrodes in the left hemisphere, whilst those defined by attenuated psychotic symptoms elicited smaller amplitudes at midline electrodes.^17^, ^18^

The auditory P300 ERP component can be separated into the P3a, associated with prefrontal activity generated by infrequent novel non‐target sounds, and the P3b, associated with temporo‐parietal activity generated by infrequent target sounds to which participants respond; both randomly presented among frequent standard sounds in 3‐stimulus versions of the classic 2‐stimulus (targets, standards) auditory oddball task.^19^ P3b attenuation has been suggested as a marker of severity for attenuated psychotic symptoms in CHR.^20^, ^21^ Moreover, attenuation in P3b but not P3a amplitude is a reliable predictor of transition to psychosis.^18^, ^20^, ^22^, ^23^ Whilst the precise relationship between P3b and basic symptoms remains unclear in CHR, findings indicate potential differences in amplitude may be present across those with and without COGDIS and may reflect diverging states of disturbed neural processing.

Rather than the specific abnormalities captured by ERPs, some argue for a broader dysfunction in neural dynamics in schizophrenia, with abnormalities both within and between distinct brain regions.^24^ Brain connectivity refers to patterns of co‐activation between distinct populations of neurons, both at rest and during tasks. Disruption in the synchronization of these patterns has been proposed as a candidate mechanism of basic symptoms.^9^ Resting‐state EEG is commonly used to examine oscillatory activity across different neural populations, and greater de‐synchronization has been reported in those with higher levels of basic symptoms.^9^ Phase synchronization is one of several methods of calculating brain connectivity and refers to the consistency of the phase‐difference between EEG nodes for specific EEG frequencies across trials. Regions that fire with consistent phase‐difference across trials are in‐phase and believed to have greater connectivity than those regions with highly variable phase‐difference across trials.^25^

Converging evidence from separate research domains point to a model of brain network dysfunction underlying the abnormal cognitive and perceptual experiences found in basic symptoms^26^, ^27^ and inter‐related constructs such as self‐disorder and depersonalization.^10^, ^28^ These models specifically argue in favor of abnormal dynamics between the ‘task‐related’ frontal–parietal network, the ‘self‐reflective’ default mode network, and sub‐cortical brain structures. Whilst resting‐state connectivity provides a representation of the brain's normal functioning, it is unable to provide insight into the coordination of different brain networks when presented with a task. To address this limitation, task‐related connectivity calculates connectivity at several time points and compares differences before and after stimulus presentation.

Hernández‐García, Martín‐Gómez^29^ measured the strength of phase synchronization across the cortex and investigated any relationship with a self‐report measure of self‐disorder, the Inventory of Psychotic‐like Anomalous Self‐Experiences. The authors reported greater overall brain connectivity in those with self‐disorder, as well as impaired ability to modulate the strength of brain connectivity upon presentation of a target stimulus in an auditory oddball task, termed connectivity strength modulation. However, to the best of our knowledge, there has been no investigation into the relationship between COGDIS and task‐related brain connectivity, to date.

The aim of the present study was to determine, in a large and well‐characterized sample of young people with CHR, whether individuals meeting COGDIS criteria show differences in EEG markers compared to those not endorsing COGDIS. Based on the previously cited CHR studies in much smaller samples,^9^ we hypothesized that CHR participants endorsing COGIDS criteria would show a reduction in P3b amplitude in central and parietal regions. We also hypothesized that participants also endorsing COGDIS would display reduced connectivity strength modulation.

## Materials and Methods

### Sample

Data were obtained from the third 5‐year prospective study of the NAPLS consortium (NAPLS‐3), which collected data between 2015 and 2018 at nine research centers across the United States.^30^ NAPLS‐3 was funded by the National Institute of Mental Health (NIMH) and data and/or research tools used in the preparation of this manuscript were obtained from the NIMH Data Archive (NDA). NAPLS‐3 comprised 806 participants between ages 12 and 30 years, of whom 96 were controls and 710 were help‐seeking CHR individuals referred *via* well‐established local referral sources, from health providers, educators, social services, or self‐referred following community education efforts. Participants were initially screened *via* telephone before attending an in‐person evaluation of eligibility and informed consent interview.^30^ Participants were excluded if they met criteria for current or lifetime axis I psychotic disorder, including affective psychoses, had a recorded IQ less than 70, had a history of a central nervous system disorder, or if diagnostic psychosis‐risk symptoms were clearly caused by an axis 1 disorder.^30^ All 710 met CHR criteria as determined by criteria of psychosis‐risk symptoms (COPS).^31^ COPS uses the structured interview for psychosis‐risk syndromes (SIPS) and the scale for assessment of psychosis‐risk symptoms (SOPS) to define and characterize CHR status. Further details regarding SIPS reliability and consensus procedures can be found elsewhere.^30^, ^31^, ^32^

### Measures

#### Basic Symptoms

Basic symptoms were assessed using the Cognitive Disturbances (COGDIS) subscale of the schizophrenia proneness instrument – adult version (SPI) and the schizophrenia proneness instrument – child and youth version. The COGDIS subscale is formed from nine items rated on a seven‐point severity scale according to their maximum frequency during the past 3 months. COGDIS criteria is then determined by the presence of two or more items rated with a severity of greater than or equal to three. COGDIS was developed to improve prognostic accuracy for risk of transition to psychosis in CHR^9^ where cognitive basic symptoms were found to play a central role.^33^ Furthermore, data from over 300 individuals aged eight and over suggest good inter‐rater reliability and construct validity for all SPI subscales.^34^, ^35^

#### Other clinical and neuropsychological measures

SOPS positive and negative symptom severity ratings were used to derive the continuous SIPS positive (SIPS+) and negative (SIPS‐) symptom scales. SIPS+ is calculated as the sum of severity ratings for all five positive symptom items, with ratings ranging 0–6, and total scores ranging between 0 and 30. SIPS‐ is calculated as the sum of severity ratings for all six negative symptoms items, with ratings ranging 0–6, and total scores ranging between 0 and 36.

Intelligence quotient was measured using the Wechsler Abbreviated Scale of Intelligence, where mean score within a healthy population is 100 (SD = 15).^36^ Word reading ability was measured using the word reading subtest from the Wide Range Achievement Test – fifth edition, where the standardized mean score is 100 (SD = 15) in a healthy population.^37^

### EEG

#### Procedure

A two‐stimulus auditory oddball paradigm was completed at baseline. The paradigm consisted of frequent standard tones (80%) and infrequent target tones (20%) presented at 80 dB (SPL) intensity *via* Etymotic Research insert earphones in three blocks of 100 trials using a fixed pseudo‐random sequence, with a 20 s rest breaks between each block. Standards (500 Hz) and targets (1000 Hz) were 50 ms pure tones (5 ms rise/fall time) presented with a stimulus onset asynchrony jittered between 1066 and 1333 ms (mean = 1250 ms). Participants viewed an instructional cartoon and heard pre‐recorded instructions directing them to keep their eyes fixed on a cross in the center of the computer display throughout the experiment and to press a button as quickly as possible with the index finger of their preferred hand in response to target tones only. The task took just under 7 min to complete.

#### Apparatus and physiological recording

The same BioSemi ActiveTwo EEG acquisition system (https://www.biosemi.com/) was used at all NAPLS‐3 sites, with a 64‐channel electrode montage and a sample rate of 1024 Hz. A standard 10–20 system for was used to position electrodes. Data were acquired with respect to common mode sense/difference mode sense‐driven reference level electrodes (CMS/DRL) and were re‐referenced offline.

#### Data processing

Data were imported into MATLAB® (R2022b)^38^ and analyzed in EEGLAB,^39^ an open‐source MATLAB® toolbox for EEG signal analysis. Before preprocessing, EEG bridging was assessed across all cephalic channels. Reference electrode standardization technique was used to re‐reference the data to a point at infinity in accordance with recommendations for ERP and connectivity analysis.^40^ Channel locations were mapped to each individual and a leadfield matrix was generated using a three‐concentric‐sphere head model. Data were filtered with a bandpass range 0.1 to 50 Hz. To remove signal drift and reduce the risk of signal distortion,^41^ we used the source information flow toolbox to perform linear piecewise detrending.^42^ We performed artifact substance reconstruction for automated artifact rejection, which uses a 0.5 s sliding window with 50% overlap and performs principal component analysis to decompose each EEG channel and identify components. Components exceeding five standard deviations within a given window were then removed. Bad channels were defined as those with activity probability limits exceeding five standard deviations and were removed. Finally, epochs were time‐locked to auditory target stimulus onsets, segmented at intervals of 1000 ms pre‐stimulus and 1000 ms post‐stimulus. An 11 ms delay was present across the Yale University and University of California San Diego sites and corrected for during epoch selection. Trials were considered correct if a participant responded to the stimulus within the accepted post‐stimulus window of 0 to 1000 ms. Participants with less than six bridged channels, five or less bad channels, and more than 50% of correct target trials were included in the analysis. On average, the percentage of correct target trials amounted to 91%.

### Electrophysiological Measures

#### P3b amplitude

Epochs were baseline corrected on 400 ms prior to stimulus onset. Participant ERP averages were calculated as the mean value of the ±50 ms window surrounding the maximum peak amplitude value captured between 200 and 400 ms post‐stimulus.^18^ A review of P3b research in schizophrenia suggests dysfunction is limited to central and temporo‐parietal electrodes,^13^ reflecting effortful “top‐down” attentional shifting towards target stimuli which require a response. This paper aims to investigate P3b alongside a brain network model of basic symptoms, in which the task‐related fronto‐parietal network (approximate to P3 and P4) may be distinct from the midline default mode network (approximate to Cz). Consequently, we calculated P3b at three electrode locations; Cz, P3, and P4.

#### Brain Connectivity—Connectivity Strength Modulation (CSM)

To calculate CSM with an EEG model of brain‐based networks, we replicated the calculations of Hernández‐García, Martín‐Gómez.^29^ To calculate neural coupling, we selected the phase‐locking value (PLV), an undirected measure of the consistency of phase‐difference between EEG signals from two electrodes, evaluating the randomness of phase difference across successive trials.^43^, ^44^, ^45^ To minimize the effects of volume conduction, a surface Laplacian spatial filter was applied, prior to calculating PLV. This aims to remove volume conducted signal from a single source but common across multiple electrodes and has been shown to reduce false positives in EEG connectivity studies.^46^, ^47^, ^48^, ^49^ We used Hilbert transform to consider the phase information from each trial^50^ and filtered signals across five frequency bands; Delta (0.5–4 Hz), Theta (4–8 Hz), Alpha (8–13 Hz), Beta (13–30 Hz), and Gamma (30–45 Hz), with increased resting connectivity strength previously reported in schizophrenia.^51^ PLV values ranged between 0 and 1, with a value of 0 indicating the phase‐difference between two EEG nodes across trials is purely random, while a value of 1 signifies that the phase difference is perfectly consistent across trials.^44^, ^45^ We calculated a global cortical CSM value for each participant by subtracting a mean pre‐stimulus connectivity strength value averaged across all electrode pairings from a mean post‐stimulus connectivity strength value. We selected a pre‐stimulus window of 0‐300 ms before onset and a post‐stimulus window of 100‐400 ms after onset.

### Statistical Analyses

Samples were split into two groups, those meeting COGDIS criteria (COGDIS+) and those not meeting criteria (COGDIS‐). To explore differences between these groups, a Pearson's chi‐squared test was used to explore variation across sex, handedness, medication, and transition rate. A Fischer's exact test was used to explore variation across ethnicity and at‐risk‐mental‐state groups. The Kolmogorov–Smirnov test was used to test normality for age, intelligence, reading ability, depression, SIPS+, SIPS‐, and stress. For age, depression, and stress, which were not normally distributed, a Wilcoxon's signed rank sum test was used to explore variation. For intelligence, SIPS+, SIPS‐, and reading ability, which were normally distributed, analysis of variance (ANOVA) was used to explore variation.

Assumptions of homogeneity and multicollinearity were checked before multiple linear regression analysis was used to investigate associations. We regressed P3b amplitude on COGDIS group, age, SIPS+, SIPS‐, and depression severity at electrode locations Cz, P3, and P4. We then regressed CSM on COGDIS group, age, SIPS+, SIPS‐, and depression severity. In both analyses, age, SIPS+, SIPS‐, and depression severity were included as covariates to control for potential confounding effects caused by differences across COGDIS−/+ groups, or because confounding effects have previously been reported in the literature.^22^, ^23^, ^52^, ^53^

**Fig. 1:**
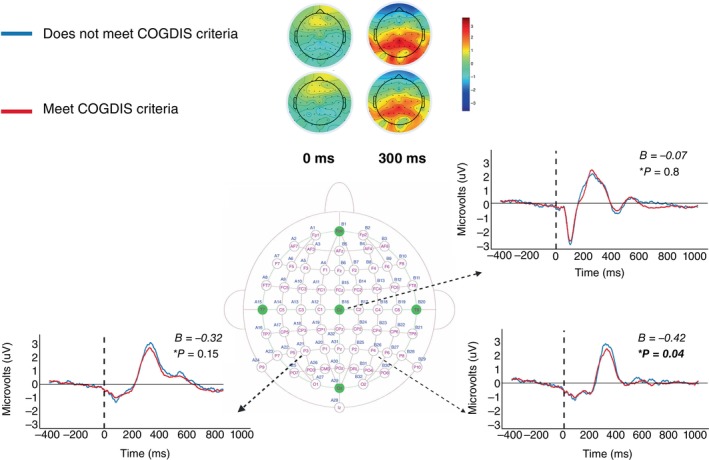
Results of the P3b analysis for clinical‐high risk (CHR) individuals who met or did not meet cognitive disturbances (COGDIS) criteria according to the schizophrenia proneness instrument. At the top, scalp topoplots show neural activity measured in μV for both groups at the time of the stimulus (0 ms) and at 300 ms post‐stimulus. Below, mean neural activity for both groups between 400 ms before stimulus and 1000 ms after stimulus are plotted for Cz, P3 and P4 electrode locations. Results of the regression analysis are listed at the top right for each electrode location. Image of 64 channel 10–20 electrode composition taken from Headcap, Headcaps. *refers to the *P*‐value of COGDIS group on p3b amplitude after correcting for multiple comparisons.

To account for multiple comparisons testing, Benjamini‐Hochburg false discovery rate correction was used when testing differences between COGDIS+/− (See Table 1 and Tables S1–S3) and in P3b analyses (See Fig. 1), adjusted *P*‐values reported. The α level was *P* = 0.05 with two‐tailed testing. Any findings beneath this threshold were considered significant and subjected to secondary analysis, where we investigated group differences across individual COGDIS items. All analyses were conducted using R 4.3.1.^54^

Ethics approval was obtained for each of the individual sites, which include Emory University, Harvard University, University of Calgary, University of California at Los Angeles, at San Diego, and at San Francisco, University of North Carolina Chapel Hill, Yale University, and Zucker Hillside Hospital. Participants were initially screened via telephone before attending an in‐person evaluation of eligibility and informed consent interview.

**Table 1:** Baseline demographics and clinical characteristics of individuals at clinical high risk for psychosis (CHR) who met (COGDIS+) or did not meet COGDIS criteria (COGDIS−)

Variable	Does not meet COGDIS criteria, *n* = 118^a^	Meets COGDIS criteria, *n* = 245^a^	*P*‐value^b^
Sex at Birth (Female)	50%	42%	0.23
Age in Years	18.39 ± 3.78	18.99 ± 3.92	0.17
Ethnic Group			0.23
Unknown	0%	0.4%	
American Indian/Alaska Native	2.5%	2.0%	
Asian	14%	12%	
Black or African American	8.5%	9.4%	
Ethnic group	0%	0%	
More than one race	18%	11%	
Native Hawaiian or Pacific Islander	1.7%	0%	
White	55%	65%	
Years of Education Completed	11.14 ± 2.70	11.79 ± 3.03	0.14
Meets criteria for an At‐Risk‐Mental‐State group			0.17
Genetic risk and deterioration	1.7%	0.4%	
Attenuated positive symptoms	96%	95%	
Attenuated positive symptoms & genetic risk and deterioration	0.8%	4.1%	
Brief Intermittent positive symptoms	1.7%	0.4%	
Attenuated positive symptoms & brief intermittent positive symptoms	0%	0%	
Handedness			
Right‐Handed	100/118	198/245	0.69
Intelligence Quotient	106.04 ± 15.74	107.50 ± 15.62	0.23
Word Reading Ability	111.23 ± 15.93	110.20 ± 17.50	0.9
Medication			
No Medication	47/118	83/245	0.23
Anti‐Psychotic	33/118	58/245	
Other	37/118	101/245	
Total Depression Score	4.96 ± 4.24	7.13 ± 4.25	<0.001
Daily Stress Score	60.52 ± 48.30	73.03 ± 55.01	0.08
SIPS+	12.14	14.14	<0.001
SIPS‐	9.58	13.81	<0.001
Transitioned to Psychosis	5.9%	12%	0.17

^a^ %(n/N); Mean ± SD(N), SIPS+ = Total severity of positive symptoms from the structured interview for psychosis‐risk syndromes (P1‐P4), SIPS‐ = Total severity of negative symptoms from the structured interview for psychosis‐risk syndromes (N1‐N5).

^b^ Pearson's Chi‐squared test; Wilcoxon rank sum test; Fisher's exact test, Analysis of Variance (ANOVA), adjusted *via* FDR correction.

## Results

At baseline, 608 participants had completed the auditory oddball EEG task. Of these, 508 participants completed both the COGDIS items of the SPI and the auditory oddball task at baseline. 431 were CHR participants. After EEG preprocessing, a total of 363 participants met the inclusion criteria and were included in the analyses. All interactions met assumptions for homogeneity of slopes and cross correlations between the three electrodes suggested the signals are not highly correlated. Baseline demographics are listed in Table 1 for those included in the analysis and in Tables S1 and S2 (see supplementary material) for those excluded during EEG preprocessing.

### Event‐related potentials—P3b

Figure 1 displays the results of the multiple linear regression model analyzing the relationship between COGDIS groups and P3b amplitude across Cz, P3, and P4 electrode locations, with SIPS+, SIPS‐, age, and depression as covariates.

Meeting COGDIS criteria was associated with reduced P3b amplitude at the P4 electrode, *P* = 0.04 (see Fig. 1 and Table S1). In contrast, COGDIS criteria was not significantly associated with P3b amplitude at the Cz and P3 electrode locations (see Fig. 1 and Supplementary Table S1). When analyzing the association between COGDIS severity and P3b amplitude across all three electrode locations, our results showed the strongest association at P4, however this association was no longer significant after controlling for multiple testing (see Table S3 and Fig. S1).

Secondary analysis investigating the relationship between P3b amplitude at the P4 electrode location and individual COGDIS items did not identify any associations (see Table S4).

### Brain Connectivity

**Fig. 2:**
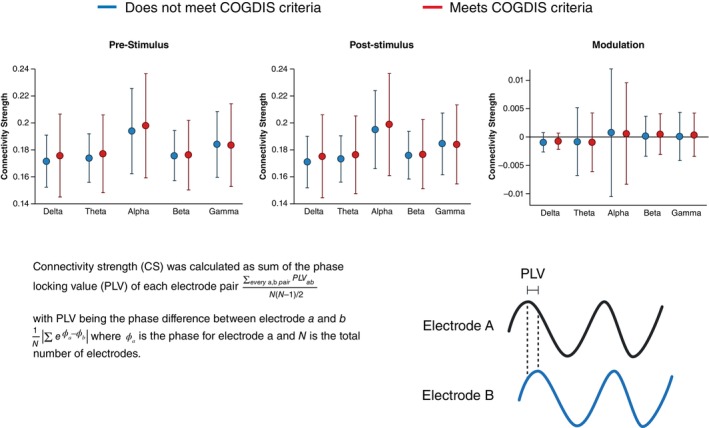
At the top, mean connectivity strength values for COGDIS+/− groups are plotted across each frequency band at each time window. At the bottom left, the equation for average connectivity strength is provided, At the bottom right, a visual representation of phase locking value (PLV) is provided.

Figure 2 shows the results of the overall cortical connectivity strength modulation analysis. Mean connectivity strength values varied across the five frequency bands, with no significant differences between the COGDIS+/− groups. Removal of spatial filtering resulted in higher overall connectivity values in both windows and across all five frequency bands (see Table S5), with no significant differences between the COGDIS+/− groups.

## Discussion

In this study, to better understand the neurophysiological basis of basic symptoms in CHR, we investigated the relationship between COGDIS and EEG features including the P3b amplitude and CSM after a target stimulus. Our results suggest that endorsing additional COGDIS criteria was associated with smaller P3b amplitude at the P4 electrode when compared to subjects who meet CHR criteria alone, whilst controlling for the severity of positive and negative symptoms, and depression. Further analysis at the item level of COGDIS did not identify any associations between P3b amplitude and item severity or endorsement, suggesting that P3b differences cannot be attributed to specific COGDIS items. COGDIS was also not associated with CSM after being presented with a target stimulus, used as an index of task‐related brain connectivity.

In temporo‐parietal regions approximate to the P4 electrode, P3b attenuation has been suggested to reflect poor memory operations.^55^, ^56^ Our results suggest this deficit may be larger in those who screen positive for COGDIS. Our findings are unlikely to be influenced by other factors known to impact P3b amplitude in an auditory oddball task, such as shorter tone duration and shorter interstimulus intervals.^13^ Although COGDIS appears to cluster in CHR populations relative to controls, it remains highly prevalent across comorbidities and can be carefully distinguished from the severity of positive and negative symptoms, perhaps indicating a transdiagnostic phenomenon which reflects a specific form of disturbed information processing in those at risk for psychosis *and* other psychiatric conditions.^57^, ^58^, ^59^, ^60^

Bayesian brain models presuppose the brain as an organ of statistical inference which acts to minimize uncertainty, by predicting future events based on past experience and sensory information.^61^ Past experience can be quantified mathematically as the prior probability of a hypothesis before evidence is available, namely in the form of memory. Bayesian models of cognitive perceptual disturbances, such as basic symptoms and self‐disorder,^61^, ^62^ argue that the degree of belief in a hypothesis is adjusted based on the likelihood the hypothesis is true, given both the sensory information and prior probability. This equation determines a posterior probability, which is consolidated in memory to update prior probability. In this context, P3b amplitude can be viewed as indexing sensory precision, with lower P3b amplitude reflecting greater reliance on incoming sensory data.^63^, ^64^ The predictive coding model of psychosis suggests that psychotic symptoms can arise from both increased and reduced prior precision,^65^, ^66^ with weak priors often accompanied by heightened sensory precision, potentially linked to increased dopamine activity and hypofunction of N‐Methyl‐D‐Aspartate receptors.^65^, ^67^ Phenomenologically, patients would present with an over‐preoccupation with the sensory world and difficulties discriminating salient from non‐salient stimuli in the perceptual landscape. One of the COGDIS items, thought insertion, has been theorized to develop in precisely this way^68^ and our findings suggest that individuals meeting COGIDS criteria display reduced P3b, further supporting the role of excessive sensory precision in early psychotic states.

Our results show no evidence of CSM differences across COGDIS+/− groups. Previous studies utilizing this method have reported a deficit in the modulation of functional connectivity in schizophrenia^51^ and one study investigating self‐disorder in a cohort of 25 persons with chronic or first episode schizophrenia, reported a significant deficit in CSM in those exhibiting self‐disorder.^29^ In our analysis we used an additional spatial filter to minimize potential spurious connectivity. Removal of spatial filtering resulted in equivalent overall connectivity strength and CSM values but had no effect on the differences between groups (see Fig. S1). Differences in the examined samples may explain these findings. Compared to healthy controls, measurable CSM differences may emerge at a point where chronic or first‐episode schizophrenia is already established, rather than in group comparisons within pre‐psychotic NAPLS‐3 CHR cohort. In any event, our results indicate that EEG measures of CSM may *not* be a useful paradigm for the purpose of clinical stratification in CHR.

This research was a secondary analysis of NAPLS‐3, and we acknowledge several limitations of our study. Two central limitations of secondary analysis research are whether the researchers were privy to study‐specific nuances that may affect their analysis and whether the measures were collected with the hypothesis in mind.^69^ Firstly, we were able to access study specific information and addressed this limitation by controlling for these nuances in our analysis, though we acknowledge that certain measures were not available for all participants. Secondly, we acknowledge that in the NAPLS‐3 protocol, the two‐tone auditory oddball task was not the primary paradigm and was included for the purpose of harmonizing ERP paradigms across NAPLS3 and several other CHR consortia and was chosen because auditory target P3b had been shown in prior studies to predict conversion to psychosis.^18^, ^22^, ^23^ Similarly, the COGDIS items of the SPI were not a primary clinical measure and were incorporated as an adjunct phenomenological screening tool. For example, only COGDIS items from the SPI‐A/CY were collected as part of the NAPLS‐3 protocol, whilst cognitive perceptual (COPER) basic symptoms, which complement COGDIS, were not recorded. These dichotomous cut‐off values offer clear, actionable thresholds, which are especially valuable in early intervention contexts. However, dichotomous thresholds may overlook symptom nuance which could be better captured by the granularity of dimensional severity scales, at the risk of clear decision‐making.^70^ Furthermore, no medication differences were found between COGDIS+/− groups, though dose was not included as a co‐variate. Considering these factors alongside the *P*‐value of the P3b at P4, we advise caution in the interpretation of data, noting that data were not collected with this analysis in mind.

Critics also highlight that P3b attenuation lacks specificity for psychosis or schizophrenia spectrum disorders, and has been reported in mood disorders, obsessive‐compulsive disorder, and some neurodegenerative conditions.^71^ Moreover, we were not able to correct for factors such as smoking and substance use which can suppress P3b amplitude and are prevalent in CHR.^72^ Thus, P3b attenuation is perhaps best thought of as a transdiagnostic marker indexing higher burden of subtle cognitive‐perceptual disturbances linked to a higher risk for mental illness persistence and progression.

In summary, larger P3b attenuation was observed at the P4 electrode, approximating the temporo‐parietal region of the right hemisphere, in CHR participants additionally endorsing COGDIS, relative to CHR status alone. Whilst some have suggested P3b attenuation as a marker of illness severity in psychosis, our findings show no association with the severity of either positive or negative symptoms at Cz, P3 or P4. Regarding a model of brain network dysfunction in basic symptoms, we found no significant differences across COGDIS+/− groups in a measure of task‐related brain connectivity. Our findings suggest that COGDIS reflects distinct neurophysiological processes, separate from psychotic and depressive symptom severity, and may reflect a transdiagnostic disturbance that clusters in CHR but remains highly prevalent across CHR comorbidities. The P3b ERP, but not measures of CSM, may have translational utility in CHR samples.

## Funding

J. C. Martin is funded through the Ian Scott Scholarship, from the Australian Rotary Health Foundation (HPE CM 2023/5282). S. Hartmann is funded through the Prediction of Early Mental Disorder and Preventive Treatment (PRE‐EMPT, www.pre-empt.org.au)—Centre of Research Excellence (NHMRC grant: #1198304). D. Mathalon is funded through the US National Institute of Mental Health (U01 MH076989). S. R. Clark has participated in advisory and educational boards and received speaker's fees from Janssen‐Cliag, Lundbeck, Otsuka, and Servier; research funding Janssen‐Cilag, Lundbeck, Otsuka, and Gilead; and data sharing from Viatris Australia. K. O. Schubert has participated in advisory and educational boards and received speaker's fees from Janssen‐Cilag, Lundbeck, Otsuka, and has received research funding from Janssen‐Cilag, Lundbeck, Otsuka, and Gilead.

## Disclosure statement

We declare that we have no competing interests to disclose.

## Author contributions

J. C. Martin was involved in conceptualization, analysis, drafting manuscript. S. Hartmann was involved in conceptualization, supervision, and editing manuscript. D. Mathalon was involved in editing manuscript. S. R. Clark was involved in conceptualization, supervision, and editing manuscript. K. O. Schubert was involved in conceptualization, supervision, and editing manuscript.

## Supporting information


**Data S1.** Supporting Information.

## Data Availability

Data and/or research tools used in the preparation of this manuscript were obtained from the National Institute of Mental Health (NIMH) Data Archive (NDA). NDA is a collaborative informatics system created by the National Institutes of Health to provide a national resource to support and accelerate research in mental health. Dataset identifier(s): [10.15154/5e91‐fe76]. This manuscript reflects the views of the authors and may not reflect the opinions or views of the NIH or of the Submitters submitting original data to NDA.

## References

[1] Kyzar EJ , Denfield GH . Taking subjectivity seriously: Towards a unification of phenomenology, psychiatry, and neuroscience. Mol. Psychiatry 2023; 28: 10–16. 36460728 10.1038/s41380-022-01891-2 PMC10130907

[2] Klosterkötter J , Hellmich M , Steinmeyer EM , Schultze‐Lutter F . Diagnosing schizophrenia in the initial prodromal phase. Arch. Gen. Psychiatry 2001; 58: 158–164. 11177117 10.1001/archpsyc.58.2.158

[3] Yung AR , Wood SJ , Malla A , Nelson B , McGorry P , Shah J . The reality of at risk mental state services: A response to recent criticisms. Psychol. Med. 2021; 51: 212–218. 31657288 10.1017/S003329171900299X PMC7893503

[4] Hazan H , Spelman T , Amminger GP *et al*. The prognostic significance of attenuated psychotic symptoms in help‐seeking youth. Schizophr. Res. 2020; 215: 277–283. 31615738 10.1016/j.schres.2019.10.016

[5] Hartmann JA , Yuen HP , McGorry PD *et al*. Declining transition rates to psychotic disorder in “ultra‐high risk” clients: Investigation of a dilution effect. Schizophr. Res. 2016; 170: 130–136. 26673973 10.1016/j.schres.2015.11.026

[6] Solmi M , Soardo L , Kaur S *et al*. Meta‐analytic prevalence of comorbid mental disorders in individuals at clinical high risk of psychosis: The case for transdiagnostic assessment. Mol. Psychiatry 2023; 28: 2291–2300. 37296309 10.1038/s41380-023-02029-8 PMC10611568

[7] Martin JC , Clark SR , Hartmann S , Schubert KO . A tale of three spectra: Basic symptoms in clinical‐high‐risk of psychosis vary across autism Spectrum disorder, schizotypal personality disorder, and borderline personality disorder. Schizophr. Bull. Open 2024; 5: sgae017. 39183768 10.1093/schizbullopen/sgae017 PMC11341945

[8] Stüble M , Schultze‐Lutter F , Kaess M *et al*. Clinical and neurocognitive profiles of a combined clinical high risk for psychosis and clinical control sample: Latent class analysis. BJPsych Open. 2024; 10: e226. 39635738 10.1192/bjo.2024.815 PMC11698148

[9] Schultze‐Lutter F , Debbané M , Theodoridou A *et al*. Revisiting the basic symptom concept: Toward translating risk symptoms for psychosis into neurobiological targets. Front. Psychiatry 2016; 7: 9. 26858660 10.3389/fpsyt.2016.00009 PMC4729935

[10] Martin JC , Clark SR , Schubert KO . Towards a Neurophenomenological understanding of self‐disorder in schizophrenia Spectrum disorders: A systematic review and synthesis of anatomical, physiological, and neurocognitive findings. Brain Sci. 2023; 13: 845. 37371325 10.3390/brainsci13060845 PMC10296413

[11] Etkin A , Mathalon DH . Bringing imaging biomarkers into clinical reality in psychiatry. JAMA Psychiatr. 2024; 81: 1142–1147. 10.1001/jamapsychiatry.2024.2553 39230917

[12] Perrottelli A , Giordano GM , Brando F , Giuliani L , Mucci A . EEG‐based measures in At‐risk mental state and early stages of schizophrenia: A systematic review. Front. Psychiatry 2021; 12: 653642. 34017273 10.3389/fpsyt.2021.653642 PMC8129021

[13] Hamilton HK , Mathalon DH , Ford JM . P300 in schizophrenia: Then and now. Biol. Psychol. 2024; 187: 108757. 38316196 10.1016/j.biopsycho.2024.108757 PMC11686549

[14] Nelson B , Lavoie S , Gawęda Ł *et al*. The neurophenomenology of early psychosis: An integrative empirical study. Conscious. Cogn. 2020; 77: 102845. 31678780 10.1016/j.concog.2019.102845

[15] Sass LA , Parnas J . Schizophrenia, consciousness, and the self. Schizophr. Bull. 2003; 29: 427–444. 14609238 10.1093/oxfordjournals.schbul.a007017

[16] Parnas J , Møller P , Kircher T *et al*. EASE: Examination of anomalous self‐experience. Psychopathology 2005; 38: 236–258. 16179811 10.1159/000088441

[17] Frommann I , Brinkmeyer J , Ruhrmann S *et al*. Auditory P300 in individuals clinically at risk for psychosis. Int. J. Psychophysiol. 2008; 70: 192–205. 18700155 10.1016/j.ijpsycho.2008.07.003

[18] Hamilton HK , Roach BJ , Bachman PM *et al*. Association between P300 responses to auditory oddball stimuli and clinical outcomes in the psychosis risk syndrome. JAMA Psychiatry 2019; 76: 1187–1197. 31389974 10.1001/jamapsychiatry.2019.2135 PMC6686970

[19] Polich J , Criado JR . Neuropsychology and neuropharmacology of P3a and P3b. Int. J. Psychophysiol. 2006; 60: 172–185. 16510201 10.1016/j.ijpsycho.2005.12.012

[20] Tang Y , Wang J , Zhang T *et al*. P300 as an index of transition to psychosis and of remission: Data from a clinical high risk for psychosis study and review of literature. Schizophr. Res. 2020; 226: 74–83. 30819593 10.1016/j.schres.2019.02.014 PMC6708777

[21] Graber K , Bosquet Enlow M , Duffy FH *et al*. P300 amplitude attenuation in high risk and early onset psychosis youth. Schizophr. Res. 2019; 210: 228–238. 30685392 10.1016/j.schres.2018.12.029

[22] van Tricht MJ , Nieman DH , Koelman JHTM *et al*. Reduced parietal P300 amplitude is associated with an increased risk for a first psychotic episode. Biol. Psychiatry 2010; 68: 642–648. 20627236 10.1016/j.biopsych.2010.04.022

[23] Hamilton HK , Woods SW , Roach BJ *et al*. Auditory and visual oddball stimulus processing deficits in schizophrenia and the psychosis risk syndrome: Forecasting psychosis risk with P300. Schizophr. Bull. 2019; 45: 1068–1080. 30753731 10.1093/schbul/sby167 PMC6737543

[24] Uhlhaas PJ , Singer W . Oscillations and neuronal dynamics in schizophrenia: The search for basic symptoms and translational opportunities. Biol. Psychiatry 2015; 77: 1001–1009. 25676489 10.1016/j.biopsych.2014.11.019

[25] Bastos AM , Schoffelen JM . A tutorial review of functional connectivity analysis methods and their interpretational pitfalls. Front. Syst. Neurosci. 2015; 9: 175. 26778976 10.3389/fnsys.2015.00175 PMC4705224

[26] Andreou C , Leicht G , Nolte G *et al*. Resting‐state theta‐band connectivity and verbal memory in schizophrenia and in the high‐risk state. Schizophr. Res. 2015; 161: 299–307. 25553979 10.1016/j.schres.2014.12.018

[27] van Tricht MJ , Ruhrmann S , Arns M *et al*. Can quantitative EEG measures predict clinical outcome in subjects at clinical high risk for psychosis? A prospective multicenter study. Schizophr. Res. 2014; 153: 42–47. 24508483 10.1016/j.schres.2014.01.019

[28] Sierra M , Berrios GE . Depersonalization: neurobiological perspectives. Biol. Psychiatry 1998; 44: 898–908. 9807645 10.1016/s0006-3223(98)00015-8

[29] Hernández‐García M , Martín‐Gómez C , Gómez‐García M *et al*. Abnormal self‐experiences related to a hypersynchronic brain state in schizophrenia. Schizophr. Res. 2020; 222: 538–540. 32507377 10.1016/j.schres.2020.03.056

[30] Addington J , Liu L , Brummitt K *et al*. North American Prodrome longitudinal study (NAPLS 3): Methods and baseline description. Schizophr. Res. 2022; 243: 262–267. 32317224 10.1016/j.schres.2020.04.010 PMC7572535

[31] McGlashan T , Walsh B , Woods S . The Psychosis‐Risk Syndrome: Handbook for Diagnosis and Follow‐Up. Oxford University Press, Oxford, UK, 2010.

[32] Addington J , Cadenhead KS , Cannon TD *et al*. North American Prodrome longitudinal study: A collaborative multisite approach to prodromal schizophrenia research. Schizophr. Bull. 2007; 33: 665–672. 17255119 10.1093/schbul/sbl075 PMC2526151

[33] Schultze‐Lutter F , Ruhrmann S , Fusar‐Poli P , Bechdolf A , Schimmelmann G , Klosterkotter J . Basic symptoms and the prediction of first‐episode psychosis. Curr. Pharm. Des. 2012; 18: 351–357. 22239566 10.2174/138161212799316064

[34] Olsen KA , Rosenbaum B . Prospective investigations of the prodromal state of schizophrenia: Assessment instruments. Acta Psychiatr. Scand. 2006; 113: 273–282. 16638071 10.1111/j.1600-0447.2005.00698.x

[35] Fux L , Walger P , Schimmelmann BG , Schultze‐Lutter F . The schizophrenia proneness instrument, child and youth version (SPI‐CY): Practicability and discriminative validity. Schizophr. Res. 2013; 146: 69–78. 23473813 10.1016/j.schres.2013.02.014

[36] Wechsler D . Wechsler Abbreviated Scale of Intelligence—Second Edition (WASI‐II). APA PsycTests, San Antonio, Texas, USA, 2011.

[37] Wilkinson GS , Robertson GJ . WRAT‐5: Wide Range Achievement Test Professional Manual. Psychological Assessment Resources, Bloomington, Minnesota, USA, 2017.

[38] MATLAB . R2022b ed. Natick, Massachusetts: The MathWorks Inc. 2022.

[39] Delorme A , Makeig S . EEGLAB: An open source toolbox for analysis of single‐trial EEG dynamics including independent component analysis. J. Neurosci. Methods 2004; 134: 9–21. 15102499 10.1016/j.jneumeth.2003.10.009

[40] Yao D , Qin Y , Hu S , Dong L , Bringas Vega ML , Valdés Sosa PA . Which reference should we use for EEG and ERP practice? Brain Topogr. 2019; 32: 530–549. 31037477 10.1007/s10548-019-00707-x PMC6592976

[41] de Cheveigné A , Arzounian D . Robust detrending, rereferencing, outlier detection, and inpainting for multichannel data. Neuroimage 2018; 172: 903–912. 29448077 10.1016/j.neuroimage.2018.01.035 PMC5915520

[42] Delorme A , Mullen T , Kothe C *et al*. EEGLAB, SIFT, NFT, BCILAB, and ERICA: New tools for advanced EEG processing. Comput. Intell. Neurosci. 2011; 2011: 130714. 21687590 10.1155/2011/130714 PMC3114412

[43] Lachaux JP , Rodriguez E , Martinerie J , Varela FJ . Measuring phase synchrony in brain signals. Hum. Brain Mapp. 1999; 8: 194–208. 10619414 10.1002/(SICI)1097-0193(1999)8:4<194::AID-HBM4>3.0.CO;2-C PMC6873296

[44] Roach BJ , Mathalon DH . Event‐related EEG time‐frequency analysis: An overview of measures and An analysis of early gamma band phase locking in schizophrenia. Schizophr. Bull. 2008; 34: 907–926. 18684772 10.1093/schbul/sbn093 PMC2632478

[45] Mathalon DH , Sohal VS . Neural oscillations and synchrony in brain dysfunction and neuropsychiatric disorders: It's about time. JAMA Psychiatr. 2015; 72: 840–844. 10.1001/jamapsychiatry.2015.0483 26039190

[46] Cohen MX . Comparison of linear spatial filters for identifying oscillatory activity in multichannel data. J. Neurosci. Methods 2017; 278: 1–12. 28034726 10.1016/j.jneumeth.2016.12.016

[47] Cohen MX . Comparison of different spatial transformations applied to EEG data: A case study of error processing. Int. J. Psychophysiol. 2015; 97: 245–257. 25455427 10.1016/j.ijpsycho.2014.09.013

[48] Brunner C , Billinger M , Seeber M , Mullen TR , Makeig S . Volume conduction influences scalp‐based connectivity estimates. Front. Comput. Neurosci. 2016; 10: 121. 27920674 10.3389/fncom.2016.00121 PMC5119053

[49] Lai M , Demuru M , Hillebrand A , Fraschini M . A comparison between scalp‐ and source‐reconstructed EEG networks. Sci. Rep. 2018; 8: 12269. 30115955 10.1038/s41598-018-30869-w PMC6095906

[50] Namburi P . Phase locking value. In: Exchange, MCF. 2024.

[51] Gomez‐Pilar J , de Luis‐García R , Lubeiro A *et al*. Deficits of entropy modulation in schizophrenia are predicted by functional connectivity strength in the theta band and structural clustering. NeuroImage: Clinical. 2018; 18: 382–389. 29487795 10.1016/j.nicl.2018.02.005 PMC5814380

[52] Lee SY , Namkoong K , Cho HH , Song DH , An SK . Reduced visual P300 amplitudes in individuals at ultra‐high risk for psychosis and first‐episode schizophrenia. Neurosci. Lett. 2010; 486: 156–160. 20858531 10.1016/j.neulet.2010.09.035

[53] Perlman G , Foti D , Jackson F , Kotov R , Constantino E , Hajcak G . Clinical significance of auditory target P300 subcomponents in psychosis: Differential diagnosis, symptom profiles, and course. Schizophr. Res. 2015; 165: 145–151. 25934167 10.1016/j.schres.2015.04.013 PMC4457683

[54] Team RC . R: A Language and Environment for Statistical Computing. R Foundation for Statistical Computing, Vienna, Austria, 2023.

[55] Polich J . Updating P300: An integrative theory of P3a and P3b. Clin. Neurophysiol. 2007; 118: 2128–2148. 17573239 10.1016/j.clinph.2007.04.019 PMC2715154

[56] Polich J . Theoretical overview of P3a and P3b. In: Detection of Change: Event‐Related Potential and fMRI Findings. Springer US, Boston, MA, 2003; 83–98.

[57] Bechdolf A , Wagner M , Ruhrmann S *et al*. Preventing progression to first‐episode psychosis in early initial prodromal states. Br. J. Psychiatry 2012; 200: 22–29. 22075649 10.1192/bjp.bp.109.066357

[58] Frearson G , de Otazu OJ , Catalan A , Aymerich C , Salazar de Pablo G . Review: Efficacy of preventative interventions for children and adolescents at clinical high risk of psychosis—A systematic review and meta‐analysis of intervention studies. Child and Adolescent Mental Health 2025; 30: 66–82. 39688301 10.1111/camh.12755 PMC11754713

[59] Tiffin PA , Kelleher I . Commentary: Time to abandon the ‘clinical high risk state for psychosis’ (CHR‐P) concept in adolescence? Commentary on Frearson et al. ‘Efficacy of preventative interventions for children and adolescents at clinical high risk of psychosis: A systematic review and meta‐analysis of intervention studies’. Child Adolesc. Ment. Health 2025. 10.1111/camh.12776 40125946

[60] Koutsouleris N , Buciuman M‐O , Neuner L‐M *et al*. Refining Schizophrenia Risk Assessment: Machine Learning Delineates a Brain Signature of Cognitive Basic Symptoms. 2025. 10.21203/rs.3.rs-6278819/v1

[61] Ciaunica A , Seth A , Limanowski J , Hesp C , Friston KJ . I overthink—Therefore I am not: An active inference account of altered sense of self and agency in depersonalisation disorder. Conscious. Cogn. 2022; 101: 103320. 35490544 10.1016/j.concog.2022.103320 PMC9130736

[62] Araya JM , López‐Silva P , Rosen C . The narrative self‐model in schizophrenia: Integrating predictive processing with phenomenological psychopathology. Phenomenology Cog. Sci. 2024: 1–21.

[63] Cao R , Cao G , Liu P . Increasing perceptual salience diminishes the motor interference effect from dangerous objects. Front. Psychol. 2020; 11: 580. 32292380 10.3389/fpsyg.2020.00580 PMC7118218

[64] Kopp B , Seer C , Lange F *et al*. P300 amplitude variations, prior probabilities, and likelihoods: A Bayesian ERP study. Cogn. Affect. Behav. Neurosci. 2016; 16: 911–928. 27406085 10.3758/s13415-016-0442-3

[65] Sterzer P , Adams RA , Fletcher P *et al*. The predictive coding account of psychosis. Biol. Psychiatry 2018; 84: 634–643. 30007575 10.1016/j.biopsych.2018.05.015 PMC6169400

[66] Corlett PR , Horga G , Fletcher PC , Alderson‐Day B , Schmack K , Powers AR 3rd. Hallucinations and strong priors. Trends Cogn. Sci. 2019; 23: 114–127. 30583945 10.1016/j.tics.2018.12.001 PMC6368358

[67] Fletcher PC , Frith CD . Perceiving is believing: A Bayesian approach to explaining the positive symptoms of schizophrenia. Nat. Rev. Neurosci. 2009; 10: 48–58. 19050712 10.1038/nrn2536

[68] Sterzer P , Mishara AL , Voss M , Heinz A . Thought insertion as a self‐disturbance: An integration of predictive coding and phenomenological approaches. Front. Hum. Neurosci. 2016; 10: 502. 27785123 10.3389/fnhum.2016.00502 PMC5060939

[69] Cheng HG , Phillips MR . Secondary analysis of existing data: Opportunities and implementation. Shanghai Arch. Psychiatry 2014; 26: 371–375. 25642115 10.11919/j.issn.1002-0829.214171 PMC4311114

[70] Allardyce J , Suppes T , Van Os J . Dimensions and the psychosis phenotype. Int. J. Methods Psychiatr. Res. 2007; 16: S34–S40. 17623393 10.1002/mpr.214 PMC6879079

[71] Raggi A , Serretti A , Ferri R . The P300 component of the auditory event‐related potential in adult psychiatric and neurologic disorders: A narrative review of clinical and experimental evidence. Int. Clin. Psychopharmacol. 2024; 40: 259–274. 39163164 10.1097/YIC.0000000000000566

[72] Turetsky BI , Dress EM , Braff DL *et al*. The utility of P300 as a schizophrenia endophenotype and predictive biomarker: Clinical and socio‐demographic modulators in COGS‐2. Schizophr. Res. 2015; 163: 53–62. 25306203 10.1016/j.schres.2014.09.024 PMC4382423

