# Supplementary material for: Associations between attenuated auditory p300 event‐related potential and cognitive basic symptoms in young people at clinical high risk for psychosis
Source: Psychiatry Clin Neurosci. 2025 Aug 21;79(11):739–46. doi: 10.1111/pcn.13886 (PMC12580602; doi:10.1111/pcn.13886)
Supplement: Supplementary file 1 — Data S1. Supporting Information. [file PCN-79-739-s001.docx]

**Supplemental Material**

*Table S1: Comparing Demographics and Clinical Characteristics between CHR participants included in the primary analysis and CHR participants excluded during EEG preprocessing steps, who either met (COGDIS+) or did not meet COGDIS criteria (COGDIS−)*

| Variable | **Participants included**, N = 363^1^ | **Participants excluded**, N = 68^1^ | **p-value**^2^ |
| --- | --- | --- | --- |
| **Sex at Birth (Female)** | 45% | 41% | 0.91 |
| **Age in Years** | 18.79+/-3.88 | 19.23+/-4.23 | 0.77 |
| **Ethnic Group** |  |  | 0.99 |
| Unknown | 0% | 0% |  |
| American Indian/Alaska Native | 2% | 1% |  |
| Asian | 12% | 12% |  |
| Black or African American | 9% | 10% |  |
| White | 62% | 65% |  |
| Native Hawaiian or Pacific Islander | 0% | 0% |  |
| More than one ethnicity | 13% | 12% |  |
| **Years of Education Completed** | 11.57+/-2.94 | 11.91+/-3.09 | 0.77 |
| **Meets criteria for an At-Risk-Mental-State group** |  |  | 0.99 |
| Genetic risk and deterioration | 1% | 1% |  |
| Attenuated positive symptoms | 95% | 96% |  |
| Attenuated positive symptoms & genetic risk and deterioration | 3% | 3% |  |
| Brief Intermittent positive symptoms | 1% | 0% |  |
| Attenuated positive symptoms & brief intermittent positive symptoms | 0% | 0% |  |
| **Meets COGDIS criteria** | 245/363 | 38/68 | 0.45 |
| **Handedness** |  |  | 0.45 |
| Right-Handed | 298/363 | 52/68 |  |
| **Intelligence Quotient** | 107.02+/-15.65 | 107.29+/-13.96 | 0.99 |
| **Word Reading Ability** | 110.53+/-16.99 | 111.76+/-17.27 | 0.88 |
| **Medication** |  |  | 0.51 |
| No Medication | 130/363 | 30/68 |  |
| Anti-Psychotic | 91/363 | 20/68 |  |
| Other | 138/363 | 18/68 |  |
| **Total Depression Score** | 6.42+/-4.35 | 5.36+/-3.91 | 0.45 |
| **Daily Stress Score** | 69.06.83+/-53.22 | 70.24+/-52.01 | 0.91 |
| **SIPS+** | 13.48+/-3.94 | 12.81+/-3.63 | 0.51 |
| **SIPS-** | 12.45+/-6.27 | 11.34+/-6.60 | 0.53 |
| **Transitioned to Psychosis** | 36/363 | 6/68 | 0.99 |
| ^1^%(n/N); Mean+/-SD(N), SIPS+ = Total severity of positive symptoms from the structured interview for psychosis-risk syndromes (P1-P4), SIPS- = Total severity of negative symptoms from the structured interview for psychosis-risk syndromes (N1-N5) | | | |
| ^2^Pearson's Chi-squared test; Wilcoxon rank sum test; Fisher's exact test, Analysis of Variance (ANOVA), adjusted via FDR correction | | | |

*Table S2: Baseline Demographics and Clinical Characteristics of Individuals at Clinical High Risk for Psychosis (CHR) who were excluded in EEG preprocessing steps, but either met (COGDIS+) or did not meet COGDIS criteria (COGDIS−)*

| Variable | **Does not meet COGDIS criteria**, N = 30^1^ | **Meets COGDIS criteria**, N = 38^1^ | **p-value**^2^ |
| --- | --- | --- | --- |
| **Sex at Birth (Female)** | 53% | 32% | 0.28 |
| **Age in Years** | 18.22+/-4.17 | 20.05+/-4.16 | 0.28 |
| **Ethnic Group** |  |  | 0.58 |
| Unknown | 0% | 0% |  |
| American Indian/Alaska Native | 0% | 2.6% |  |
| Asian | 13% | 11% |  |
| Black or African American | 3.3% | 16% |  |
| White | 73% | 58% |  |
| Native Hawaiian or Pacific Islander | 0% | 0% |  |
| More than one ethnicity | 10% | 13% |  |
| **Years of Education Completed** | 11.30+/-3.39 | 12.39+/-2.80 | 0.40 |
| **Meets criteria for an At-Risk-Mental-State group** |  |  | 0.58 |
| Genetic risk and deterioration | 0% | 2.6% |  |
| Attenuated positive symptoms | 100% | 92% |  |
| Attenuated positive symptoms & genetic risk and deterioration | 0% | 5.3% |  |
| Brief Intermittent positive symptoms | 0% | 0% |  |
| Attenuated positive symptoms & brief intermittent positive symptoms | 0% | 0% |  |
| **Handedness** |  |  |  |
| Right-Handed | 23/30 | 29/38 | 0.88 |
| **Intelligence Quotient** | 105.93+/-13.08 | 108.41+/-14.72 | 0.58 |
| **Word Reading Ability** | 109.57+/-15.96 | 113.54+/-18.29 | 0.58 |
| **Medication** |  |  | 0.58 |
| No Medication | 16/30 | 14/30 |  |
| Anti-Psychotic | 8/30 | 12/30 |  |
| Other | 6/30 | 12/30 |  |
| **Total Depression Score** | 3.70+/-3.02 | 6.68+/-4.07 | **0.02** |
| **Daily Stress Score** | 82.83+/-61.01 | 59.45+/-40.71 | 0.30 |
| **SIPS+** | 12.00+/-3.29 | 13.44+/-3.80 | 0.29 |
| **SIPS-** | 8.33+/-5.99 | 13.78+/-6.11 | **0.007** |
| **Transitioned to Psychosis** | 2/30 | 4/38 | 0.89 |
| ^1^%(n/N); Mean+/-SD(N), SIPS+ = Total severity of positive symptoms from the structured interview for psychosis-risk syndromes (P1-P4), SIPS- = Total severity of negative symptoms from the structured interview for psychosis-risk syndromes (N1-N5) | | | |
| ^2^Pearson's Chi-squared test; Wilcoxon rank sum test; Fisher's exact test, Analysis of Variance (ANOVA), adjusted via FDR correction | | | |

*Table S3 – Relationship between COGDIS+/- group and P3b amplitude across Cz, P3, and P4 electrodes* (N=363)

|  | **Cz** | | | | **P3** | | | | **P4** | | | |
| --- | --- | --- | --- | --- | --- | --- | --- | --- | --- | --- | --- | --- |
| **Characteristic**^1,4^ | **Beta** | **95% CI**^2^ |  | ***P****^3^* | **Beta** | **95% CI**^2^ |  | ***P****^3^* | **Beta** | **95% CI**^2^ |  | ***P****^3^* |
| Meets COGDIS criteria | -0.07 | -0.60, 0.45 |  | 0.8 | -0.32 | -0.76, 0.12 |  | 0.15 | -0.42 | -0.82, -0.02 |  | **0.04** |
| SIPs Positive Severity | -0.03 | -0.09, 0.03 |  | 0.3 | -0.01 | -0.06, 0.04 |  | 0.7 | 0.00 | -0.05, 0.05 |  | 0.9 |
| SIPs Negative Severity | 0.01 | -0.03, 0.05 |  | 0.7 | 0.02 | -0.01, 0.05 |  | 0.2 | 0.00 | -0.03, 0.03 |  | 0.8 |
| Age | -0.03 | -0.08, 0.03 |  | 0.4 | -0.07 | -0.12, -0.03 |  | **0.003** | -0.05 | -0.09, 0.00 |  | **0.04** |
| Depression Severity | 0.06 | 0.00, 0.11 |  | **0.04** | 0.02 | -0.02, 0.07 |  | 0.4 | 0.02 | -0.03, 0.06 |  | 0.4 |
| Total Daily Stress | 0.00 | -0.01, 0.00 |  | 0.12 | 0.00 | -0.01, 0.00 |  | **0.02** | 0.00 | -0.01, 0.00 |  | 0.07 |

^1^COGDIS items were measured as endorsed/not endorsed based on COGIDS criteria, ^2^CI = Confidence Interval, ^3^p-value adjusted via FDR correction, ^4^multiple linear regression model included SIPS positive severity, SIPS negative severity, age, depression severity, and total daily stress

*Table S3 – Relationship between COGDIS severity and P3b amplitude across Cz, P3, and P4 electrodes* (N=363)

|  | **Cz** | | | | **P3** | | | | | **P4** | | | | |  |
| --- | --- | --- | --- | --- | --- | --- | --- | --- | --- | --- | --- | --- | --- | --- | --- |
| **Characteristic**^1,4^ | **Beta** | **95% CI**^2^ |  | ***P****^3^* | | **Beta** | **95% CI**^2^ |  | ***P****^3^* | | **Beta** | **95% CI**^2^ |  | ***P****^3^* | |
| Sum of COGDIS items endorsed | -0.07 | -0.60, 0.45 |  | 0.8 | | -0.06 | -0.16, 0.04 |  | 0.3 | | -0.08 | -0.17, 0.01 |  | 0.27 | |
| SIPs Positive Severity | -0.03 | -0.09, 0.03 |  | 0.9 | | -0.01 | -0.06, 0.04 |  | 0.9 | | 0..00 | -0.05, 0.05 |  | 0.9 | |
| SIPs Negative Severity | 0.01 | -0.03, 0.05 |  | 0.8 | | 0.02 | -0.01, 0.05 |  | 0.6 | | 0.00 | -0.03, 0.03 |  | 0.8 | |
| Age | -0.03 | -0.08, 0.03 |  | 0.4 | | -0.08 | -0.12, -0.03 |  | **0.006** | | -0.05 | -0.09, 0.00 |  | **0.045** | |
| Depression Severity | 0.06 | 0.00, 0.11 |  | 0.12 | | 0.02 | -0.03, 0.07 |  | 0.5 | | 0.02 | -0.03, 0.06 |  | 0.5 | |
| Total Daily Stress | 0.00 | -0.01, 0.00 |  | 0.12 | | 0.00 | -0.01, 0.00 |  | 0.09 | | 0.00 | -0.01, 0.00 |  | 0.11 | |

^1^COGDIS items were measured as endorsed/not endorsed based on COGIDS criteria, ^2^CI = Confidence Interval, ^3^p-value adjusted via FDR correction, ^4^multiple linear regression model included SIPS positive severity, SIPS negative severity, age, depression severity and total daily stress


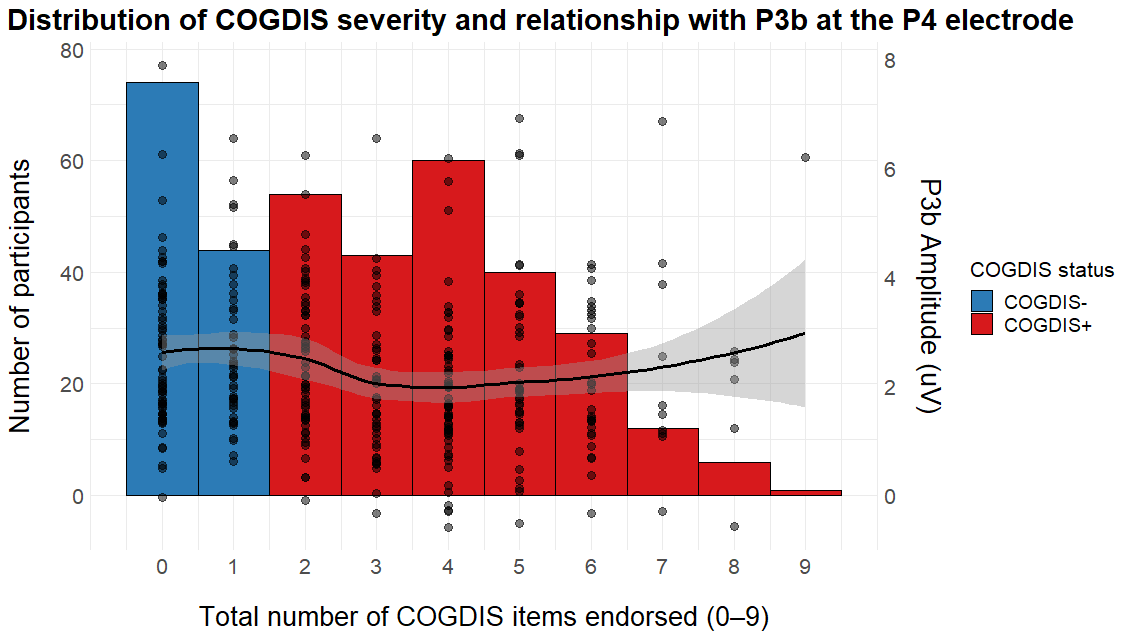
Figure S1 – Histogram of COGDIS severity distribution and scatterplot to show the relationship between total number of COGDIS items endorsed and p3b amplitude at the P4 electrode (N=363). A LOESS model of COGDIS severity and P3b amplitude, where the line represents expected values and the shaded regions represent 95% confident intervals.

*Table S4– COGDIS+/- group or COGDIS severity and P3b amplitude at P4 electrode* (N=363)

|  | **P3b amplitude at P4 electrode** | | | | | | | |
| --- | --- | --- | --- | --- | --- | --- | --- | --- |
|  | **Item Endorsed+/-** ^1^ | | | | **Item severity**^2^ | | | |
| **COGDIS item**^5^ | **Beta** | **95% CI**^3^ |  | ***P****^4^* | **Beta** | **95% CI**^3^ | | ***P****^4^* |
| Inability to Divide Attention (B1) | -0.14 | -0.53, 0.24 |  | 0.75 | 0.03 | -0.07, 0.13 | | 0.68 |
| Thought Interference (C2) | -0.09 | -0.46, 0.27 |  | 0.9 | -0.01 | -0.11, 0.10 | | 0.9 |
| Thought Blockages (C3) | -0.24 | -0.62, 0.13 |  | 0.6 | -0.08 | -0.20, 0.03 | | 0.6 |
| Disturbance in receptive speech (C4) | -0.249 | -0.71, 0.13 |  | 0.6 | -0.04 | -0.16, 0.08 | | 0.68 |
| Disturbance in Expressive Speech (C5) | -0.45 | -0.82, -0.05 |  | 0.25^6^ | -0.11 | -0.22, 0.00 | | 0.36^6^ |
| Thought Pressure (D3) | -0.12 | -0.49, 0.24 |  | 0.75 | -0.05 | -0.15, 0.05 | | 0.68 |
| Unstable Ideas of Reference (D4) | -0.04 | -0.44, 0.35 |  | 0.9 | -0.03 | -0.15, 0.08 | | 0.67 |
| Disturbances of Abstract Thinking (O3) | -0.19 | -0.75, -0.37 |  | 0.75 | -0.10 | -0.25, 0.06 | | 0.6 |
| Captivation of attention by details of the visual field (O7) | -0.03 | -0.37, 0.44 |  | 0.9 | -0.04 | -0.08, 0.15 | | 0.68 |
| ^1^COGDIS items were measured as endorsed/not endorsed based on COGIDS criteria, ^2^COGDIS items were measured using a severity scale (0-7), ^3^CI = Confidence Interval, ^4^p-value adjusted via FDR correction, ^5^multiple linear regression model included SIPS positive severity, SIPS negative severity, age, depression severity, and stress, ^6^p-value <0.05 prior to FDR correction | | | | | | |  |  |

*Table S5- Associations between COGDIS+/- and Connectivity strength without surface Laplacian spatial filtering*

| Frequency Band^4^ | Connectivity Strength^1^ | | | | |  | |  |
| --- | --- | --- | --- | --- | --- | --- | --- | --- |
|  |  | **Mean**  **(+/-_SD)** |  |  | **Cohen's d^2,3^** | | **p-value** | |
| Delta | Pre-stimulus^5^ | 0.30963  (+/- 0.05) |  |  | 0.052 | | 0.63 | |
|  | Post-Stimulus^5^ | 0.32746  (+/- 0.05) |  |  | 0.011 | | 0.92 | |
|  | Modulation^6^ | 0.01784  (+/- 0.018) |  |  | 0.172 | | 0.14 | |
| Theta | Pre-stimulus | 0.30912  (+/- 0.05) |  |  | 0.052 | | 0.63 | |
|  | Post-Stimulus | 0.32676  (+/- 0.05) |  |  | 0.011 | | 0.92 | |
|  | Modulation | 0.01764  (+/- 0.018) |  |  | 0.172 | | 0.14 | |
| Alpha | Pre-stimulus | 0.30761  (+/- 0.05) |  |  | 0.052 | | 0.63 | |
|  | Post-Stimulus | 0.32470  (+/- 0.04) |  |  | 0.010 | | 0.92 | |
|  | Modulation | 0.01709  (+/- 0.017) |  |  | 0.173 | | 0.14 | |
| Beta | Pre-stimulus | 0.29664  (+/- 0.05) |  |  | 0.052 | | 0.64 | |
|  | Post-Stimulus | 0.31014  (+/- 0.04) |  |  | 0.002 | | 0.99 | |
|  | Modulation | 0.01350  (+/- 0.014) |  |  | 0.167 | | 0.15 | |
| Gamma | Pre-stimulus | 0.26963  (+/- 0.06) |  |  | 0.008 | | 0.94 | |
|  | Post-Stimulus | 0.27027  (+/- 0.06) |  |  | 0.006 | | 0.95 | |
|  | Modulation | 0.00064  (+/- 0.006) |  |  | 0.015 | | 0.89 | |

| ^1^Connectivity strength measured as the average phase-locking value across cortical electrodes, averaged across a specified time window, ^2^ANCOVA model of connectivity strength by COGDIS criteria, controlling for SIPS positive severity, SIPS negative severity, age, depression severity, and stress, ^3^ COGDIS = endorsed/not endorsed based on COGIDS criteria, ^4^Frequency bands in Hz: Delta[0.5-4], Theta[4-8], Alpha[8-13], Beta[13-30], Gamma[30-45], ^5^Time windows in *ms* surrounding auditory stimulus: Pre-stimulus [-300 to 0], Post-stimulus [0-300], ^6^Modulation = Post-stimulus – Pre-stimulus |
| --- |
